# Effect of stomach size on organs at risk in pancreatic stereotactic body radiotherapy

**DOI:** 10.1186/s13014-022-02107-1

**Published:** 2022-07-31

**Authors:** Osamu Tanaka, Takuya Taniguchi, Kousei Adachi, Shuto Nakaya, Takuji Kiryu, Akira Ukai, Chiyoko Makita, Masayuki Matsuo

**Affiliations:** 1 Department of Radiation Oncology, Asahi University Hospital, 3-23 Hashimoto-cho, Gifu City, Gifu 500-8523 Japan; 2 Department of Oral and Maxillofacial Surgery, Asahi University Hospital, 3-23 Hashimoto-cho, Gifu City, Gifu 500-8523 Japan; 3 Department of Radiology, Gifu University Hospital, 1-1 Yanagido, Gifu City, Gifu 501-1194 Japan

**Keywords:** Radiotherapy, Pancreas, Organ at risk, Stereotactic body radiotherapy, Stomach

## Abstract

**Background:**

In clinical practice, the organs at risk (OARs) should be carefully determined when performing pancreatic stereotactic body radiotherapy (SBRT). We conducted a simulation study to examine the effect of the stomach size on the radiation dose to the OARs when performing pancreatic SBRT.

**Methods:**

Twenty-five cases were included in this study. Pancreatic head and body tumors were 2-cm-sized pseudotumors, which were included as gross target volume (GTV) contours. The stomach, pancreas, small intestine, liver, kidneys, and spinal cord were considered as the OARs. The prescription dose for planning target volume (PTV) was 40 Gy/5fx, and the dose limit for the OARs was determined. The dose to X% of the OAR volume at X values of 0.1, 5.0, and 10.0 cc (DX) and the percentage of the OAR volume that received more than X Gy were recorded.

**Results:**

In terms of the radiation dose to the pancreatic body tumors, the stomach size was positively correlated with a dose of D10cc [correlation coefficient (r) = 0.5516) to the stomach. The r value between the radiation dose to the pancreatic head tumor and the stomach size was 0.3499. The stomach size and radiation dose to the head and body of the pancreas were positively correlated (pancreatic head D10cc: r = 0.3979, pancreatic body D10cc: r = 0.3209). The larger the stomach, the larger the radiation dose to the healthy portion of the pancreas outside the PTV.

**Conclusions:**

When performing pancreatic SBRT, the dose to the OARs depends on the stomach size. Reducing the dose to the stomach and pancreas can be achieved by shrinking the stomach.

## Background

Evidence of the effectiveness of stereotactic body radiotherapy (SBRT) for pancreatic cancer has been recently published [1–5]. Usually, a total irradiation of 50–60 Gy is divided into 25–30 doses of 1.8–2 Gy five times a week for 5–6 weeks, whereas in SBRT, one dose of 6–25 Gy, which is larger than the normal irradiation dosage, is administered 1–6 times [6–8]. A retrospective study in the United States reported that patients with locally advanced unresectable pancreatic cancer had longer median survival (13.9 months) than those undergoing chemotherapy or conventional irradiation [1]. The incidence of adverse events tends to increase if the amount of radiation per dose is high, and the determination of the optimal divided dose and number of divided doses may be an issue in the future [9].

Intensity-modulated radiation therapy (IMRT) is clinically applied to patients with pancreatic cancer to reduce the dose to the gastrointestinal tract, kidney, and liver and increase the dose to the primary lesion. The frequency of acute adverse events of grade 3 or higher can reportedly be reduced by using a dose per fraction of 1.8–2.4 Gy and a total dose of 45–60 Gy [2]. We believe that the significance of increasing the dose in IMRT will be reported in the future.

SBRT and IMRT have undergone multiple changes, which have affected their accuracy. However, in clinical practice, we not only consider the dose to the pancreas but also the dose to the organs around the pancreas, such as the kidney, small intestine, biliary system, and stomach, which are vulnerable to the organ at risk (OAR) radiation; therefore, the radiation dosage should be carefully calculated.

Among the abdominal organs, the only parameters that can be input into the device used for administering radiation before radiation therapy are the volumes of the stomach and gallbladder (both are subject to dietary restrictions, etc.). To the best of our knowledge, there is no report on pancreatic SBRT settings considering the condition of the stomach. Therefore, we conducted a simulation study to examine the effect of the stomach size on the dose to the OARs when performing pancreatic SBRT.

## Methods

### Eligibility

From January to December 2021, 25 patients who had undergone abdominal computed tomography (CT) examinations at our hospital were included in the study. The study was a simulation study that used only information that was already unlinkable and anonymized and did not require approval by the Institutional Review Board. Patients weighing between 45 and 70 kg and having no underlying disease (cirrhosis, diabetes, and history of abdominal surgery) were selected. As the tumors were 2-cm-sized pseudotumors, patients with atrophy of the head or body of the pancreas involving an area of ≤ 2 cm were excluded.

### Contouring

The pancreatic head and body tumors were 2-cm-sized pseudotumors and were included as GTV contours. Pancreatic cancer is often found in sizes larger than 2 cm. However, if the pancreatic pseudotumors are set large, the tumor may stick to nearby organs (bile duct, small intestine, and metastatic lymph node). Especially, when it is attached to the small intestine, it becomes difficult to evaluate the dose (Dmax). Therefore, we set it to 2 cm to prevent surrounding infiltration. The stomach, pancreas (whole pancreas minus GTV), small intestine (including duodenum), liver, kidneys, and spinal cord were set as OARs. The radiation oncologist performing the contouring and medical physicist formulating the treatment plan were blinded to the patients’ data. The GTV included the entire tumor and clinical tumor volume (CTV) included the GTV along with a 3-mm margin around it. The planning target volume (PTV) was the same as the CTV.

### Dose

The prescription dose for PTV was 40 Gy/5fx (5 days). The dose limit for the OARs (pancreas, stomach, kidney, liver, and small intestine) is shown in Table 1 (similar to the limit proposed by Goldsmith et al. [3]).

**Table 1:** Organ at risk (OAR) dose constraints applied for 40 Gy per five fractions in pancreatic SBRT Pancreatic clinical tumor volume (CTV) and planning target volume (PTV) of > 95% of 40 Gy in five fractions. (PTV = CTV)

OAR	D0.1 cc	D5.0 cc	D10.0 cc	V12 Gy	V21 Gy	V30 Gy	V40 Gy
Stomach	≤ 22 Gy		≤ 16 Gy	≤ 12 cc	≤ 5 cc	≤ 1 cc	
Intestine	≤ 22 Gy	≤ 17 Gy	≤ 11 Gy		≤ 5 cc	≤ 1 cc	
Pancreas				≤ 50%			> 95%
Liver		Mean dose < 15 Gy					
Left kidney		Mean dose < 10 Gy					
Right kidney		Mean dose < 10 Gy					
Spinal cord	≤ 22 Gy					≤ 1 cc	

The stomach sizes ranged from small to large (median, 290 cc; range, 132–623 cc; standard deviation, 161 cc). We investigated how SBRT to the head and body of the pancreas was associated with the dose to the OARs depending on the size of the stomach.

### Planning

The treatment plans were formulated by medical physicists with 15 and 10 years of experience. One medical physicist formulated a treatment plan for 15 and 10 cases of cancers of the head and body of the pancreas, respectively, and the other formulated a treatment plan by randomly selecting 10 cases of cancer of the head and 15 cases of cancer of the body of the pancreas. The medical physicists were blinded to each other’s treatment plans.

The treatment was approved by a radiation oncologist based on the treatment plan.

Within the dose limits stated in Table 1, the dose to X% of the OAR volume at X values of 0.1, 5.0, and 10.0 cc (DX) and the percentage of the OAR volume that received more than X Gy (VX) were recorded.

### Statistical analysis

The associations between the parameters were presented using a scatter plot matrix together with Pearson’s correlation coefficients. We evaluated 25 cases and formulated one case each for the head and body of the pancreas (total number of plans, 50). The objective variable was the stomach size (pancreas), and the explanatory variable was the value of D10.0 cc. We primarily investigated the correlation coefficient between the two variables. We used BellCurve for Excel ([version 3.22], Tokyo, Japan) for statistical analysis.

## Results

**Fig. 1:**
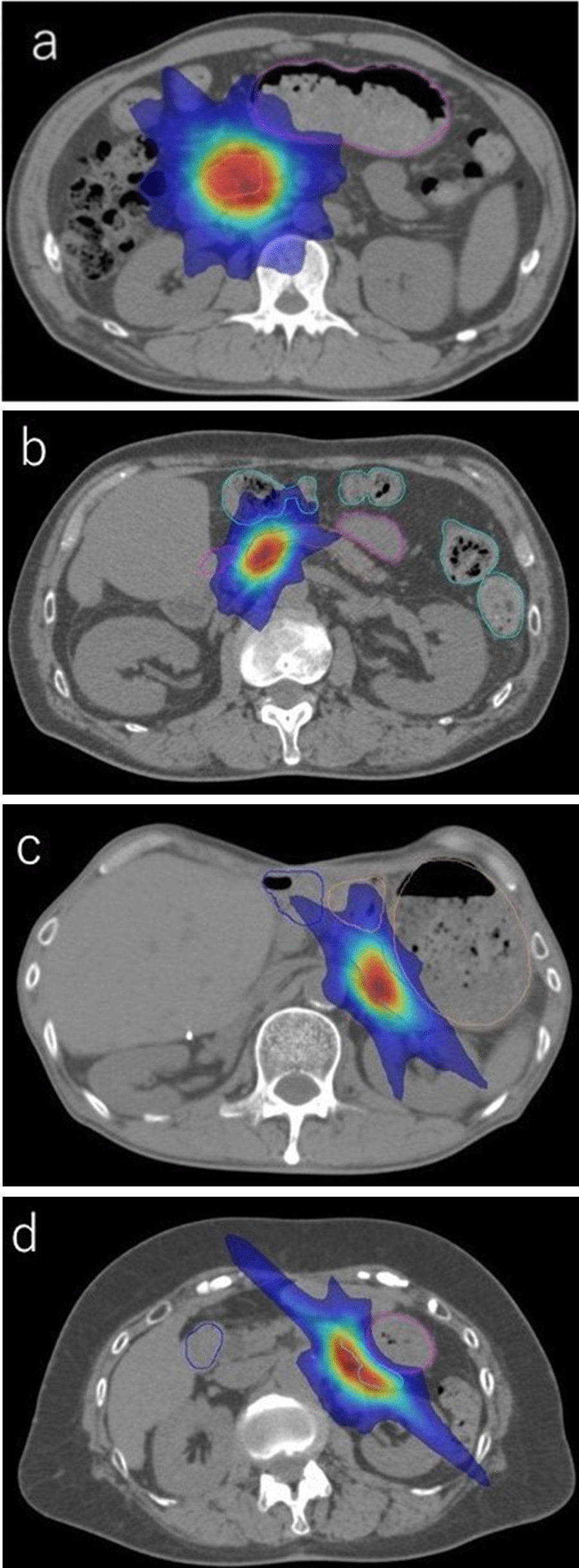
The ranges in which 100%, 110%, and 50% of the prescribed dose (40 Gy) were administered are marked in yellow, red, and blue, respectively. The positional relationship between the tumor and organs at risk (OARs) is important because the dose restrictions to the stomach and small intestine (duodenum) are strict. **a** Pancreatic head tumor and large stomach. As the head of the pancreas may be far from the stomach, even a large stomach can be administered the prescribed dose to the planning target volume (PTV). However, the duodenum will be irradiated. **b** Pancreatic head tumor and small stomach. The stomach may be distant from the tumor, but the duodenum will be irradiated. **c** Pancreatic body tumor and large stomach. A large stomach may cause the stomach to stick to the pancreas, reducing tumor PTV coverage at the site of contact with the stomach. **d** Pancreatic body tumor and small stomach. As the area in contact with the pancreas is small and may be located far from the tumor, the prescribed dose can be prepared within the limits of those to the OARs

The median stomach size was 290 cc (range, 132–623 cc). The pancreatic pseudotumors’ head tumors (Fig. 1a, b) were located far away from the stomach but were anatomically close to the duodenum, whereas the pancreatic pseudotumors’ body tumors (Fig. 1c, d) were located close to the stomach (regardless of stomach size).

Table 2 shows the D0.1 cc, D5 cc, D10 cc, V12, V15, and V21 related to PTV and OAR (pancreas, stomach, kidney, liver, and small intestine). DX and VX were separately measured for the parenchymal organs (liver, right kidney, left kidney, pancreas, and spinal cord) and luminal organs (small intestine and stomach). Except for the pancreas, the parenchymal organs did not receive high radiation doses, and the PTV of the pancreas, small intestine, and stomach required detailed examinations. In case of high radiation doses to the luminal organs, there is a risk of perforation and gastrointestinal ulcers. The correlation coefficient was calculated using D10.0 cc as it is an index that is easy to use clinically.

**Table 2:** D0.1 cc, D5cc, D10cc, V12, V15, and V21 related to PTV and OAR (pancreas, stomach, kidney, liver, and small intestine)

Pancreatic head		Mean (SD)	Pancreatic body		Mean (SD)
PTV	D0.1 cc	48.5 (2.2)	PTV	D0.1 cc	49.2 (0.9)
	D5 cc	35.3 (9.1)		D5 cc	36.7 (9.3)
	D10 cc	27.8 (10.3)		D10 cc	29.0 (9.5)
	V12 Gy	100 (0)		V12 Gy	99.9 (0.3)
	V15 Gy	99.6 (0.6)		V15 Gy	99.4 (0.9)
	V21 Gy	94 (7.0)		V21 Gy	92.2 (17.4)
Stomach	D0.1 cc	15.3 (4.3)	Stomach	D0.1 cc	19.2 (1.6)
	D5 cc	8.7 (4.0)		D5 cc	11.3 (2.3)
	D10 cc	6.8 (3.6)		D10 cc	* 9.0 (2.7)
	V12 Gy	1.2 (1.5)		V12 Gy	2.0 (1.8)
	V15 Gy	0.2 (0.5)		V15 Gy	0.3 (0.7)
	V21 Gy	0.0		V21 Gy	0.0
Intestine	D0.1 cc	18.7 (2.3)	Intestine	D0.1 cc	0.0
	D5 cc	10.1 (3.5)		D5 cc	7.7 (3.9)
	D10 cc	6.7 (4.5)		D10 cc	5.7 (3.9)
	V12 Gy	7.8 (11.4)		V12 Gy	2.1 (5.0)
	V15 Gy	2.7 (5.1)		V15 Gy	0.2 (0.6)
	V21 Gy	0.0		V21 Gy	0.0
Pancreas	D0.1 cc	48.1 (2.5)	Pancreas	D0.1 cc	48.3 (2.3)
	D5 cc	27.3 (14.6)		D5 cc	33.7 (11.4)
	D10 cc	16.0 (15.1)		D10 cc	22.0 (13.4)
	V12 Gy	40.0 (16.7)		V12 Gy	64.6 (17.7)
	V15 Gy	35.8 (15.7)		V15 Gy	57.1 (15.5)
	V21 Gy	31.3 (14.4)		V21 Gy	45.4 (15.6)
Liver	D0.1 cc	13.4 (6.9)	Liver	D0.1 cc	13.0 (9.1)
Left kidney	D0.1 cc	7.0 (3.6)	Left kidney	D0.1 cc	14.9 (8.5)
Right kidney	D0.1 cc	8.8 (3.6)	Right kidney	D0.1 cc	3.8 (1.8)
Spinal cord	D0.1 cc	6.9 (2.6)	Spinal cord	D0.1 cc	5.3 (2.1)

The correlation coefficient was calculated using D10cc as it is an index that is easy to use in clinical settings

PTV, planning target volume; DX, [the dose to X% of the volume of the organ at risk (OAR)] at X values of 0.1, 5.0, and 10.0 cc; VX (the percentage of the OAR volume that received more than X Gy)

*Coefficient of correlation r > 0.5

### Dose to the stomach

**Fig. 2:**
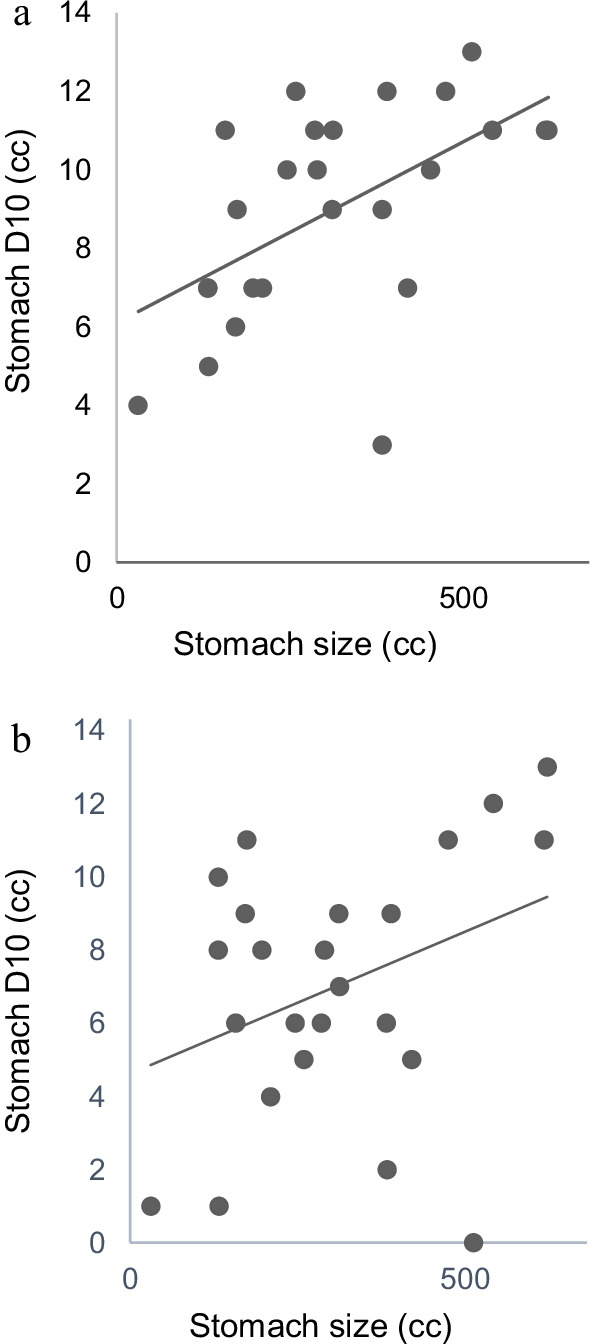
**a** Correlation between stomach size and D10 (cc) to the stomach in stereotactic body radiotherapy (SBRT) for pancreatic body tumors r = 0.5516. **b** Correlation between stomach size and D10 (cc) to the stomach in SBRT for pancreatic head tumors r = 0.3499

Overall, the dose to the stomach was higher for pancreatic body tumors than for pancreatic head tumors. In terms of the radiation dose to the pancreatic body tumors, Fig. 2a, b show that the stomach size was positively correlated with the dose of D10 cc (r = 0.5516) to the stomach. The r value between the radiation dose to the pancreatic head tumor and the size of the stomach was 0.3499.

### Dose to the pancreas

**Fig. 3:**
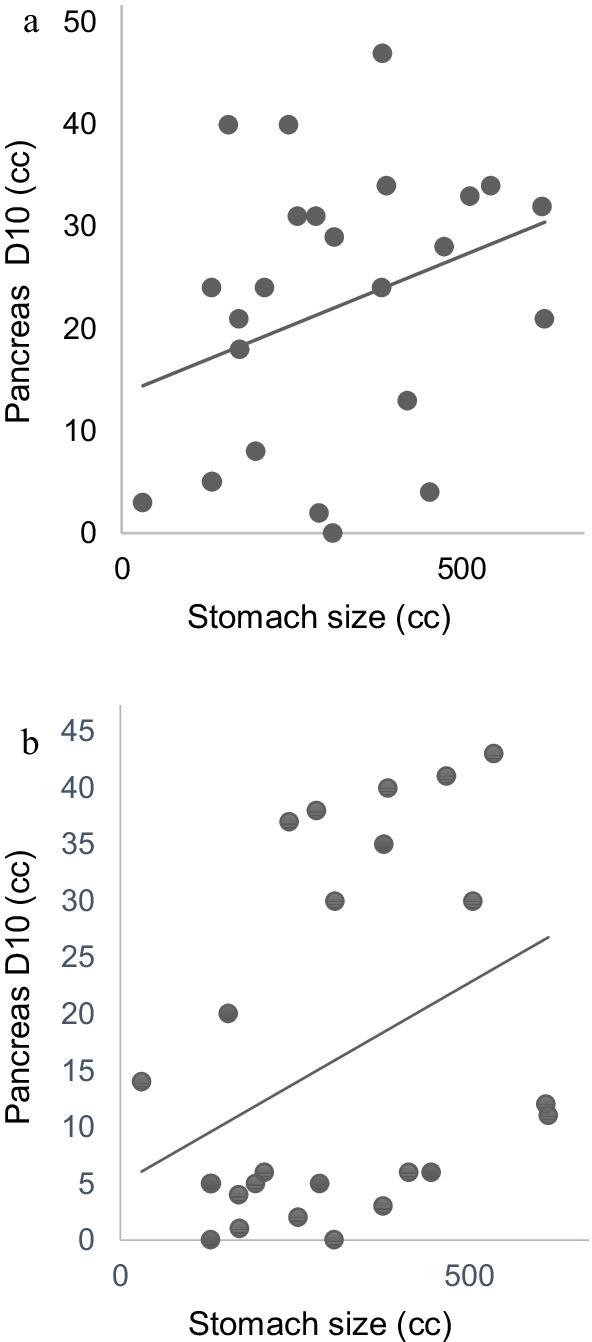
**a** Correlation between stomach size and D10 (cc) to the pancreas in SBRT for pancreatic body tumors r = 0.3209. **b** Correlation between stomach size and D10 (cc) to the pancreas in SBRT for pancreatic head tumors r = 0.3679

There was a positive correlation between the stomach size and radiation dose to the head and body of the pancreas (pancreatic head D10 cc: r = 0.3679 and pancreatic body D10 cc: r = 0.3209 (Fig. 3a, b). The larger the stomach, the larger was the dose to the pancreas (both pancreatic head and body).

### Dose to the intestine

The head of the pancreas is in contact with the duodenum and is generally higher (less correlated).

### Dose to the liver, bilateral kidneys, and spinal cord

These organs are distant from the pancreas and stomach. Therefore, they did not tend to conflict with the restricted dose (Table 2).

## Discussion

Surgically defined locally advanced pancreatic cancer (LAPC) is considered unresectable, but there is no evidence of distant metastasis. Therefore, chemotherapy is used as a first-line therapy for systemic control and local therapies, such as radiation therapy, are considered as the next step. Because local tumor control is important, a combination of chemotherapy and radiation therapy is the current standard of care for patients with LAPC. Chemoradiotherapy for local progression can improve pancreatic pain, obstructive symptoms, and quality of life in many patients. However, conventional chemoradiotherapy usually takes 6–7 weeks to complete and involves a risk of acute and late toxicity; therefore, topical treatments with greater efficacy and shorter treatment periods should be developed [2, 4, 10].

### Requirements for highly reproducible treatment setting

1. Position of PTV and OAR: Loi et al. reported that anatomical interfraction variations lead to increases in the OAR dose during SBRT for the routine imaging of pancreatic cancer using integrated CT/CyberKnife and may allow the implementation of strategies to reduce the risk of OAR over-radiation during pancreatic SBRT [5].
2. Respiratory motion control: Campbell et al. compared two competing exercise management methods: abdominal compression (AC) and the respiratory gating of pancreatic SBRT. The reduction owing to compression was significant in the anterior–posterior/up–down direction, but the decrease owing to gating was significant in all directions. Respiratory gating also showed better coverage in scenarios with reduced margins. Respiratory gating is the most effective strategy for reducing pancreatic SBRT movement and may allow the administration of increased doses of radiation through reduced target margins [11]^.^
3. Combined use of images [magnetic resonance imaging (MRI)]: Tyagi et al. reported that AC is a viable option for treating patients with LAPC with ablative doses on MRI-guided radiation therapy systems. However, intrafraction motion management is critical and can result in gastrointestinal OARs moving into high dose PTV areas [12].
4. Manipulating the shape of organs (the stomach): As a characteristic of the stomach, the size and morphology of the stomach can vary based on drinking water. In addition, by keeping the amount of water consumed constant in the stomach, the positional relationships of the abdominal organs with each other can be made similar each time. The morphology of the stomach in particular changes depending on the amount of gas and water in the stomach, even during irradiation [13–15].

In our simulation study, we found a positive correlation between the stomach size and dose to the OARs (stomach and pancreas). As the dose to the intestinal tract leads not only to ulcer formation but also to severe intestinal perforation, it is necessary to reduce D 0.1 cc to the maximum possible extent [16].

Therefore, we propose the following methods:

1. Maintain the morphology at the time of cone beam computed tomography (CBCT) and at the time of irradiation of the stomach with butylscopolamine [13];
2. Perform irradiation in the morning on an empty stomach [13];
3. Identify the positional relationship of the stomach with other organs by placing a gold marker in the pancreas [6];
4. Correctly identify the position of the gastrointestinal organs using magnetic resonance (MR)-Linac [17].

In this study, a simulation was performed using a patient with normal weight. People with obesity have high amounts of visceral fat; hence, the stomach and pancreas may be separated. There is a need to examine whether the gastric pretreatment administered to patients with obesity is necessary. It is also necessary to consider the case of a thin person. If the amount of visceral fat is large, it may be possible to separate the OAR from the target organ, and the reduction of weight before irradiation may theoretically be possible. However, it may be unrealistic owing to the progression of cancer.

We found a positive correlation between the stomach size and dose to the pancreas. A large stomach often means that there is food residue in the stomach, and the duodenum may be similarly large. If the organs surrounding the pancreas are large, the pancreas would be small and compressed (data not shown). Therefore, the pancreatic tumor may be in close proximity to the normal part of the pancreas. Nevertheless, we did not find an effect of irradiation on OARs other than the stomach and pancreas in this study. Considering this, the aforementioned noninvasive pretreatment may be beneficial.

The number of indications for SBRT is expected to increase in the future. Establishing a method to protect OARs by using a minimum prescribed dose is necessary [7]. There are techniques for artificially moving the position of OARs (sometimes PTV) to exclude the heart from the irradiation field; for example, respiratory synchronization in breast conserving therapy of left breast cancer is performed to reduce the radiation dose to the heart. In addition, techniques for three-dimensional alignment on the body surface have been used [18]. MR-Linac has made it possible to understand information regarding the surface and the deeper parts of the body; however, there is a lack of such devices in the market. Therefore, it may be useful to assess whether the organ position can be controlled by pretreatment.

### Limitations to this study

This was a simulation study in which highly accurate methods were combined. We previously reported that butylscopolamine could be used on an empty stomach for hemostatic irradiation for gastric cancer and that the shape of the stomach would be similar on CBCT. We formulated a similar hypothesis for pancreatic cancer; however, pancreatic cancer tends to metastasize and may not be similar to gastric cancer. In this regard, it is necessary to investigate the correlation between gastric volume and dose to the stomach in patients with pancreatic cancer [8].

Our approach clarified a clinical methodology in terms of clinical medical physics. In the future, pancreatic tumors will move toward radiomics analysis using imaging biological features (CT, MRI, and nuclear medicine) [19]. It is considered that adverse events can be predicted from the state of the pancreatic tumor before the treatment and the image changes in the pancreatic tumor and the OARs after the treatment. It requires prospective large-scale clinical trials.

## Conclusions

The incidence of pancreatic cancer is increasing, and the condition is increasingly detected at the stage of locally advanced cancer without distant metastasis via various diagnostic methods. The efficacy and safety of pancreatic SBRT has also been established. While administering SBRT, it is important to artificially separate the OAR from the target. The only organ that can be artificially moved is the stomach, and reducing the stomach size led to a reduction in the dose to the stomach and pancreas. Therefore, the condition of the stomach should be considered while administering pancreatic SBRT.

## Data Availability

Research data are stored in an institutional repository and will be shared upon request to the corresponding author.

## Abbreviations

SBRT: Stereotactic body radiotherapy
OAR: Organ at risk
GTV: Gross target volume
PTV: Planning target volume
IMRT: Intensity-modulated radiation therapy
CT: Computed tomography
LAPC: Locally advanced pancreatic cancer
AC: Abdominal compression
CBCT: Cone beam computed tomography
MR: Magnetic resonance
